# CpG oligonucleotide activates Toll-like receptor 9 and causes lung inflammation in vivo

**DOI:** 10.1186/1465-9921-8-72

**Published:** 2007-10-09

**Authors:** Pascal Knuefermann, Georg Baumgarten, Alexander Koch, Markus Schwederski, Markus Velten, Heidi Ehrentraut, Jan Mersmann, Rainer Meyer, Andreas Hoeft, Kai Zacharowski, Christian Grohé

**Affiliations:** 1 Department for Anesthesiology and Intensive Care Medicine, University Hospital Bonn, Sigmund-Freud-Strasse 25, 53125 Bonn, Germany; 2 Molecular Cardioprotection & Inflammation Group, Department of Anesthesia, Bristol Royal Infirmary, Bristol BS2 8HW, UK; 3 Molecular Cardioprotection & Inflammation Group, Department of Anesthesia, University Hospital Düsseldorf, Moorenstrasse 5, 40225 Düsseldorf, Germany; 4 Institute of Physiology II, University Hospital Bonn, Wilhelmstrasse 31, 53111 Bonn, Germany; 5 Department of Internal Medicine, University Hospital Bonn, Sigmund-Freud-Strasse 25, 53125 Bonn, Germany

## Abstract

**Background:**

Bacterial DNA containing motifs of unmethylated CpG dinucleotides (CpG-ODN) initiate an innate immune response mediated by the pattern recognition receptor Toll-like receptor 9 (TLR9). This leads in particular to the expression of proinflammatory mediators such as tumor necrosis factor (TNF-α) and interleukin-1β (IL-1β). TLR9 is expressed in human and murine pulmonary tissue and induction of proinflammatory mediators has been linked to the development of acute lung injury. Therefore, the hypothesis was tested whether CpG-ODN administration induces an inflammatory response in the lung via TLR9 *in vivo*.

**Methods:**

Wild-type (WT) and TLR9-deficient (TLR9-D) mice received CpG-ODN intraperitoneally (1668-Thioat, 1 nmol/g BW) and were observed for up to 6 hrs. Lung tissue and plasma samples were taken and various inflammatory markers were measured.

**Results:**

In WT mice, CpG-ODN induced a strong activation of pulmonary NFκB as well as a significant increase in pulmonary TNF-α and IL-1β mRNA/protein. In addition, cytokine serum levels were significantly elevated in WT mice. Increased pulmonary content of lung myeloperoxidase (MPO) was documented in WT mice following application of CpG-ODN. Bronchoalveolar lavage (BAL) revealed that CpG-ODN stimulation significantly increased total cell number as well as neutrophil count in WT animals. In contrast, the CpG-ODN-induced inflammatory response was abolished in TLR9-D mice.

**Conclusion:**

This study suggests that bacterial CpG-ODN causes lung inflammation via TLR9.

## Background

Acute lung injury (ALI) or its severe form, the acute respiratory distress syndrome (ARDS) remains a major health problem. Recent studies have estimated the incidence of these conditions to be between 15 and 34 cases per 100,000 inhabitants per year showing an overall mortality rate of 30–40% [1-3]. Depending on the underlying etiologies ARDS can be differentiated into a direct (pulmonary) and an indirect (extrapulmonary) form (for details see [4]).

ALI/ARDS are quite common in patients with sepsis [5] and sepsis-associated ARDS carries the highest mortality rates. Despite advances in the supportive care and mechanical ventilation strategies of ALI/ARDS, mortality rates remain unacceptably high [6-8]. As the pathophysiology of the disease is not fully understood, the treatment remains mainly supportive [9-13].

Experimental models of sepsis show that bacteria and bacterial cell components induce the expression of inflammatory mediators in various tissues as well as in the blood stream [14-17]. Among these mediators, proinflammatory cytokines are regarded as a major cause for the development of organ dysfunction during sepsis [18,19].

Bacterial DNA can initiate an innate immune response via Toll-like receptor 9 (TLR9) potentially leading to septic shock [20,21], septic arthritis [22], or meningitis [23]. The bacterial genome, compared to vertebrate DNA, contains a higher frequency of unmethylated cytosine-phosphate-guanine (CpG) dinucleotides. Small oligodeoxynucleotides (ODN) with unmethylated CpG dinucleotides (CpG-ODN) are able to perfectly mimic the immunostimulatory activity of bacterial DNA since bacterial DNA and synthetic oligodeoxynucleotides share similar base sequences and bind to the same receptor system (TLR9) [24-26].

The identification of TLRs has been a major advance in the understanding of the pathogenesis of septic shock [27]. To date, 13 TLRs (TLR1-13) have been described and TLR2 and TLR4 are the best-characterized receptors so far [28,29]. TLR2 detects gram-positive bacterial cell wall components, while TLR4 can recognize cell wall components of gram-negative bacteria [30,31].

Little is known about the role of TLR9 in the lung, but constitutive expression levels have been detected in human and mouse lung endothelial cells and mouse RAW264.7 cells. High TLR9 expression levels have been found in lung tumors [15,32,33]. Others have shown that CpG-ODN contributes to local inflammation of the lung following intratracheal instillation [32,34]. However, to our knowledge nothing is known regarding systemic effects of CpG-ODN and pulmonary inflammation. Therefore, we injected bacterial DNA intraperitoneally to answer the question whether bacterial DNA induces lung inflammation in a TLR9-dependent manner.

## Methods

### Animals

TLR9-deficient (TLR9-D) mice [25], back-crossed onto a C57BL/6 background were handled according to the principles of laboratory animal care (NIH publication No. 86-23, revised 1985) and experimental procedures were approved by the German government ethical and research boards (50.203.2-BN 43, 28/01).

### SIRS Model

The standard protocol for stimulation consisted of D-galactosamine sensitization (D-GalN; Roth, Karlsruhe, Germany) intraperitoneally (i.p.) with 1 mg/kg. 30 min later, mice received i.p. either 1 mL/kg saline (sal) or 1 nmol/g CpG-ODN (Thioat 1668; containing a "CG-motif": 5'-TCC-ATG-A**CG**-TTC-CTG-ATG-CT; TibMolBiol, Berlin, Germany). The stimulatory dose of 1 nmol/g BW was chosen according to earlier studies [20,21,25], which was sufficient to induce clinical symptoms of sepsis. Organs were harvested at 1, 2, 4 and 6 hours after stimulation with CpG-ODN. Unless otherwise stated in the manuscript groups consisted of 5 animals. In control experiments, stimulation with D-GalN alone for up to 6 hrs did not influence the mRNA expression of TNF-α, IL-1β and IL-6 detected by RNase Protection Assay.

Additional experiments were carried out injecting CpG-ODN intratracheally to further understand its effect during lung inflammation. Intratracheally, CpG-ODN was administered at a dose of 1 nmol/g BW. After intratracheal administration, lung myeloperoxidase, cytokine expression and leukocyte count were studied.

### Real-Time PCR for TLR9

Total RNA from murine tissue was isolated with the guanidinum thiocyanate method [35]. RNA concentration was determined by absorbance at 260 nm. Until further processing, RNA was dissolved in 100 μL of RNase-free water and stored at -80°C. Reverse transcription was performed using QIAGEN Omniscript Reverse Transcription kit (Qiagen, Hilden, Germany) according to the manufacturer's protocol. 1 μg RNA was used in 20 μL reaction mixtures containing 2 μL 10× Reverse Transcription Buffer, 2 μL dNTP mixture (5 mM of each dNTP), 1 μL Omniscript Reverse Transcriptase and 2 μL oligo-dT primers. The specific pre-made TaqMan^®^Gene Expression Assays (Applied Biosystems, Foster City, CA, USA) for murine TLR9 (Mm00446193 m1, amplicon length: 60 bp) and murine GAPDH (Mm999999915 q1) as housekeeping gene were used in this study. Real-time PCR was performed according to the manufacturer's protocol. 100 ng of single-stranded cDNA was mixed with supplied 2 × TaqMan Universal Master Mix (PN 4304437, Applied Biosystems, Foster City, CA, USA) and 1 μL of TaqMan^®^Gene Expression Assay to a final volume of 10 μl in a 384-well optical reaction plate. Each sample underwent 40 cycles of amplification in a 384-well optical reaction plate on an ABI PRISM^® ^Sequence Detection Systems (Applied Biosystems, Foster City, CA, USA). Relative quotients (RQ) of TLR9 gene expression comparing control mice with stimulated mice at different time-points were calculated with SDS Software 2.2 (Applied Systems, Foster City, CA, USA). RQ results were analyzed with GraphPad Prism 4.05 (GraphPad Software, San Diego, USA).

### Western Blot Analysis for TLR9

Tissue cells were lysed in ice-cold buffer (150 mM NaCl, 50 mM Tris-HCl, pH 7.4, 1 mM EDTA, 5 μg/mL Leupeptin, 5 μg/mL aprotinin, 1 mM PMSF, 0.1% SDS, 1% sodium deoxycholate, 1% Triton X-100) as previously published [36]. After brief centrifugation (16.800 g), supernatants were removed, total protein was determined (bicinchoninic acid method), separated by SDS-PAGE and blotted onto nitrocellulose membranes. The blots were incubated with anti-TLR9-antibody (1:1,000, IMG-431, Imgenex San Diego, CA, USA) at 4°C overnight. Horseradish peroxidase (HRP)-conjugated anti-rabbit secondary antibody (1:3,000, GE Healthcare Europe, Braunschweig, Germany) was used. Signals were visualized by enhanced chemiluminescence.

### Pulmonary nuclear and cytoplasmic extraction

Pulmonary protein extracts were prepared with NE-PER™ Nuclear and Cytoplasmic Extraction Reagents (Perbio, Bonn, Germany) according to the manufacturer's protocol [37].

### Electrophoretic mobility shift assay (EMSA)

NFκB oligonucleotides were end-labeled with [γ-32P] ATP. Binding reactions (25 μL total) were performed with nuclear extracts and the specificity of the DNA-protein binding was determined by cold chase analysis as well as with supershift assays. Nuclear extracts were incubated with 2 mg of polyclonal anti-p50 or anti-p65 antibody. DNA-protein complexes were electrophoresed, gels were dried, exposed overnight and scanned with a phosphoimager (FLA3000, Fuji film Europe, Düsseldorf, Germany ).

### Ribonuclease protection assay

Pulmonary RNA was extracted with the guanidinium thiocyanate method [35]. The mRNA-expression was determined with an RNase protection assay system [16].

### Pulmonary TNF-α and IL-1β protein expression

Pulmonary tissue was homogenized and incubated on ice for 5 min in 1 mL of ELISA buffer containing PBS, Triton X-100 (1 μL/mL), PMSF (250 mM in isopropanol, 1 μL/mL) and protease inhibitors. Samples were incubated on ice for 20 min, homogenized and centrifuged for 15 min at 4°C. TNF-α and IL-1β were determined in the supernatant using ELISA (R&D systems, Minneapolis, MN, USA).

### Plasma Cytokine Levels

Blood samples for plasma cytokine levels were obtained by cardiac puncture. Plasma levels of TNF-α, IL-1β and IL-6 (Mouse Cytokine multi-Plex for Luminex™ laser, BioSource Europe, Nivelles, Belgium) were determined using the microsphere array technique (Luminex 100 system, Luminex Corp., Austin, TX, USA) as previously described [36].

### Lung Myeloperoxidase (MPO)-Assay

The MPO-Assay was performed as previously described [38] with some minor modifications. Data are expressed as % of controls.

### Bronchoalveolar lavage (BAL) and cell counts

BAL was performed as described elsewhere [39]. Briefly, 4 h after CpG-ODN application, control- and TLR9-D mice were anaesthetized with isoflurane (Forene^®^; Abbott GmbH, Wiesbaden, Germany), and a midline incision was made to expose the trachea. An 18-G catheter was inserted into the trachea, and the lungs were lavaged two times with 500 μL PBS. Approximately 50–70% of the instilled volume was retrieved. All samples were kept on ice until processed. Total and differential cell counts in BAL fluid were determined. Subpopulations of leukocytes were determined using as hemocytometer.

### Leukocyte count

Lung tissue was fixed in 4% paraformaldehyde over night, embedded in paraffin and cut into 5 μm sections. Hematoxylin and Eosin (H&E) staining was performed using standard protocols and leukocyte accumulation was quantified. A total of ten microscopic fields covering 1 mm^2 ^were photographed and leukocytes were counted by a blinded investigator.

### Statistical Evaluation

All values are expressed as mean ± SEM. One-way or two-way ANOVA followed by Bonferroni-corrected post-hoc analysis was used when appropriate. T-test was applied for analysis of cell counts from bronchoalveolar lavage. Significant differences were considered to exist at p ≤ 0.05.

## Results

### Clinical manifestation of inflammation

Clinical symptoms of inflammation were monitored after CpG-ODN application in WT and TLR9-D mice. 2 hrs after CpG-ODN challenge, WT mice developed shock-like symptoms including ruffled hair, eye exudates, and lethargy, while TLR9-D mice were not affected.

### Pulmonary gene and protein expression of TLR9

**Figure 1:**
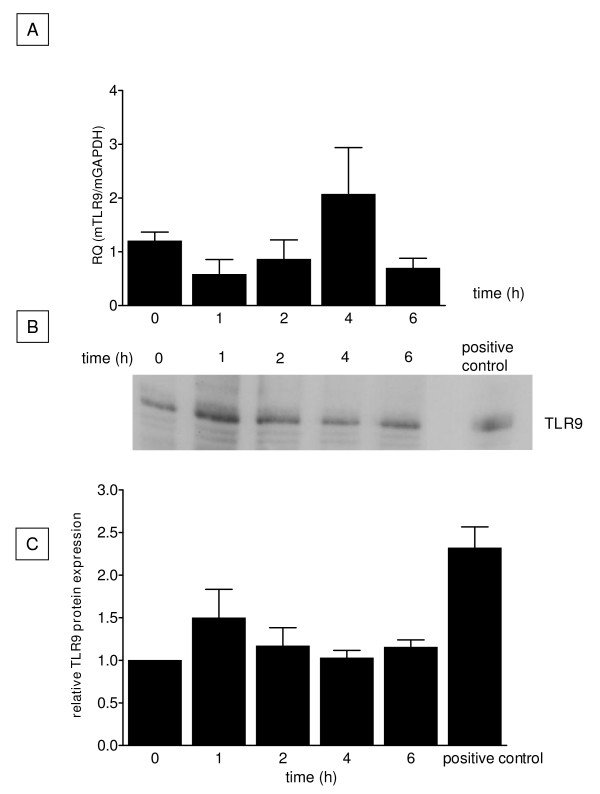
**Pulmonary expression of TLR9**. TLR9 expression in the lung was detected by Real-time PCR (A) and by Western blot analysis (B, C). All data were normalized to control (0 h) (C). TLR9 was present even under base line conditions; however, no significant increase in TLR9 was observed after CpG-ODN stimulation (n = 3/group).

The expression of TLR9 in whole native pulmonary tissue was demonstrated using Real-time PCR and Western-blot analysis. Both techniques showed a constitutive expression of TLR9 (Figure 1A–C). However, neither the mRNA nor the protein expression pattern significantly changed after agonist treatment with CpG-ODN (up to 6 hrs).

### NFκB activation in the lung after CpG-ODN stimulation

**Figure 2:**
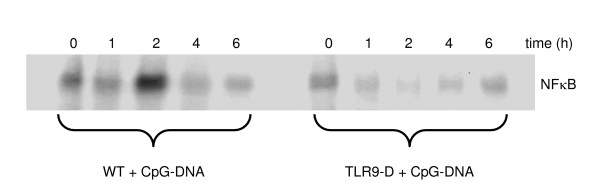
**Activation of NFκB in the lung**. A strong increase in pulmonary NFκB-DNA binding activity was observed in WT mice within 2 hrs after stimulation with CpG-ODN, whereas there was only a reduced NFκB-DNA binding activity in TLR9-D mice detectable by EMSA.

Systemic CpG-ODN treatment led to a time-dependent (maximum at 2 hrs) substantial activation of pulmonary NFκB in WT mice. In contrast, this effect was not detectable in TLR9-D mice (Figure 2).

### Pulmonary cytokine mRNA expression after CpG-ODN challenge

**Figure 3:**
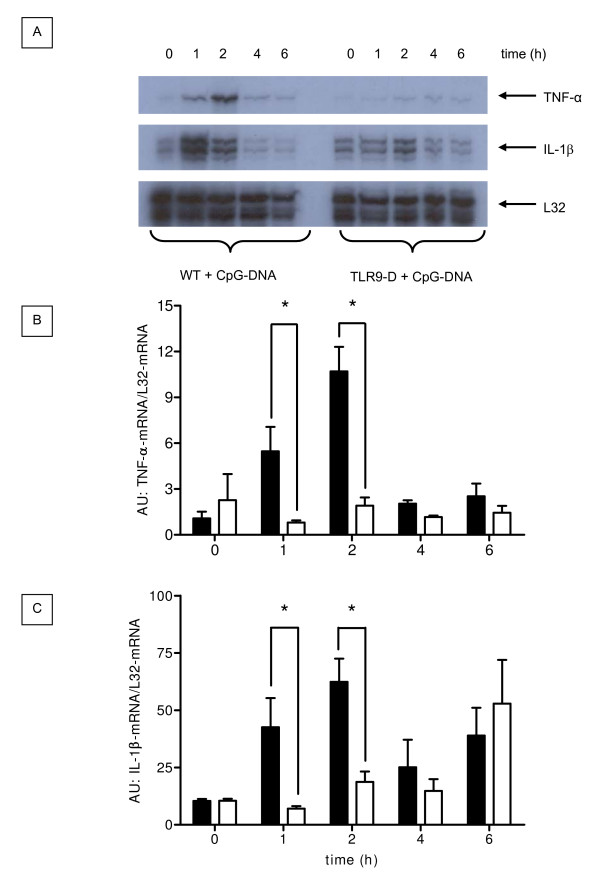
**Pulmonary proinflammatory cytokine gene expression**. Time course of pulmonary proinflammatory cytokine gene expression of TNF-α and IL-1β and the house keeping gene L32 following CpG-ODN stimulation in WT and TLR9-D mice. Densitometric analysis of the RNase Protection Assays revealed significant increases of TNF-α-mRNA/L32-mRNA (B) and IL-1β-mRNA/L32-mRNA (C) in WT mice at 1 hr and 2 hrs compared to TLR9-D animals (mean ± SEM; * *p *< 0.05; AU = arbitrary units).

CpG-ODN induced a rapid and robust increase in TNF-α and IL-1β mRNA transcripts in lungs of WT mice (Figure 3A). Densitometry (Figures 3B and 3C) revealed that peak cytokine expression occurred 2 hrs after injection of CpG-ODN and was not present in TLR9-D mice (p ≤ 0.05).

### Pulmonary cytokine protein expression following CpG-ODN challenge

**Figure 4:**
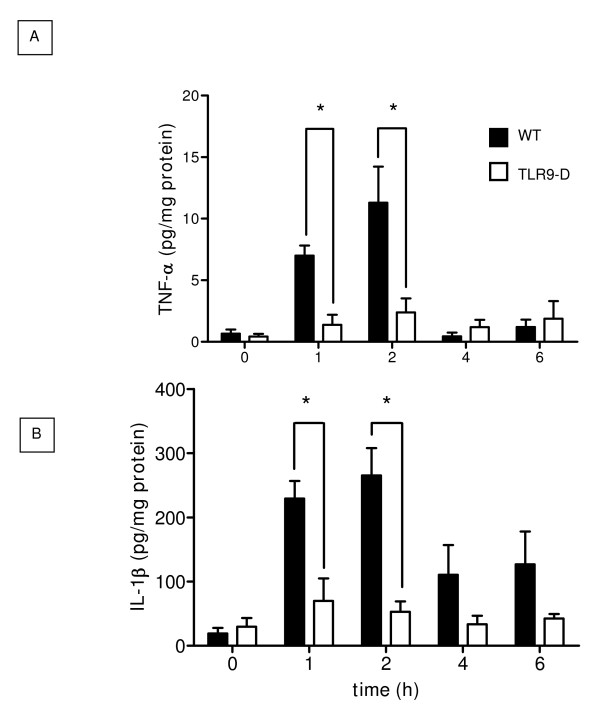
**Expression of pulmonary TNF-α and IL-1β protein**. Expression of pulmonary TNF-α (A) and IL-1β (B) detected by ELISA in WT and TLR9-D mice at different time points following CpG-ODN stimulation. Results were normalized to total protein content of lung tissue. A maximum in cytokine production was observed 2 hrs after CpG-ODN challenge. TNF-α (A) and IL-1β (B) protein expression were significantly higher in WT compared to TLR9-D mice (mean ± SEM; * *p *< 0.05).

To determine whether increased mRNA expression paralleled also increased cytokine protein levels in the lung, we tested the *in vivo *induction of TNF-α and IL-1β protein expression in WT and TLR9-D mice by ELISA. Figures 4A and 4B illustrate that CpG-ODN administration led to a significant increase in protein expression of TNF-α and IL-1β in pulmonary tissue from control mice. A significant increase in cytokine production can be observed 1 hr after injection of CpG-ODN with a peak protein expression at 2 hrs. At 2 hrs, TNF-α and IL-1β protein levels were significantly higher in WT compared to TLR9-D mice. Figures 4A and 4B show that the kinetics of TNF-α and IL-1β protein production parallels the up-regulation of the corresponding mRNA-transcripts.

To exclude solely extrapulmonary effects of CpG-ODN on the lung, WT- and TLR9-D mice received CpG-ODN also intratracheally. This route of administration again resulted in lung inflammation, e.g. demonstrated by a significant cytokine response in WT animals. 2 hrs after CpG-ODN challenge, pulmonary TNF-α tissue levels were significantly increased in WT mice (7.0 ± 0.6 pg/mg tissue) when compared to TLR9-D animals (0.6 ± 0.2 pg/mg tissue; *p *< 0.05). Also IL-1β levels were significantly raised in WT mice (62 ± 12 pg/mg tissue) when compared to TLR9-D animals (16 ± 1 pg/mg tissue; *p *< 0.05).

### Plasma cytokine levels following CpG-ODN challenge

**Figure 5:**
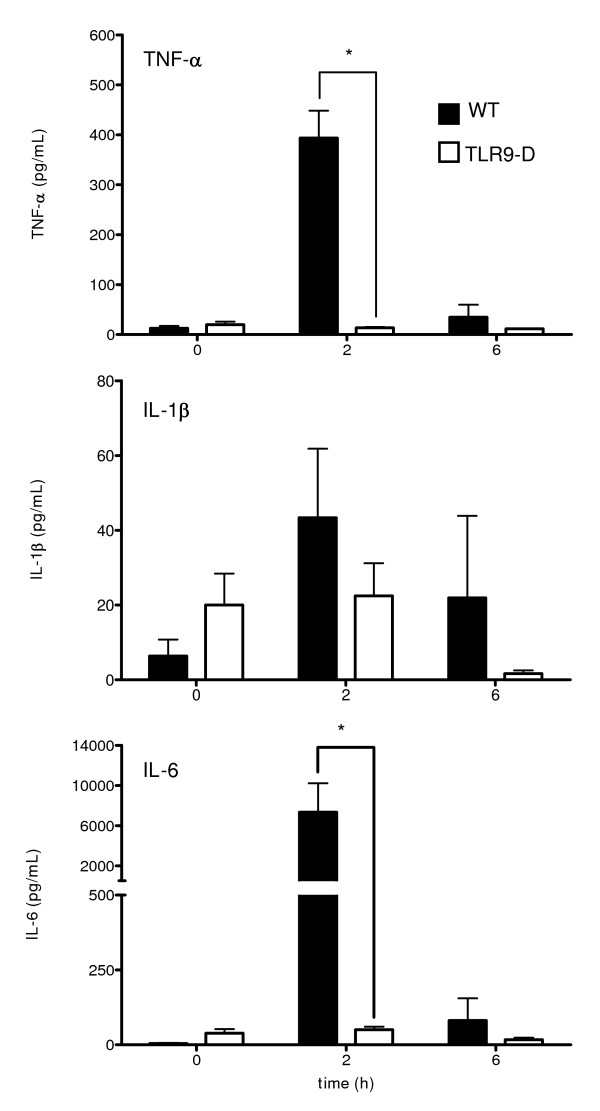
**Plasma cytokine levels**. Plasma levels of TNF-α, IL-1β and IL-6 were determined using the microsphere array technique. CpG-ODN led to a significant increase in plasma cytokine levels of TNF-α and IL-6 within 2 hrs (mean ± SEM; * *p* < 0.05).

CpG-ODN-treated WT animals showed a significant increase in the plasma levels of the cytokines TNF-α and IL-6 after 2 hrs. Similarly, plasma levels of IL-1β increased as well after 2 hrs without reaching statistical significance. These effects were not detectable in CpG-ODN-treated TLR9-D mice (Figure 5). After 6 hrs, cytokine levels in WT mice return to baseline levels.

### MPO activitiy

**Figure 6:**
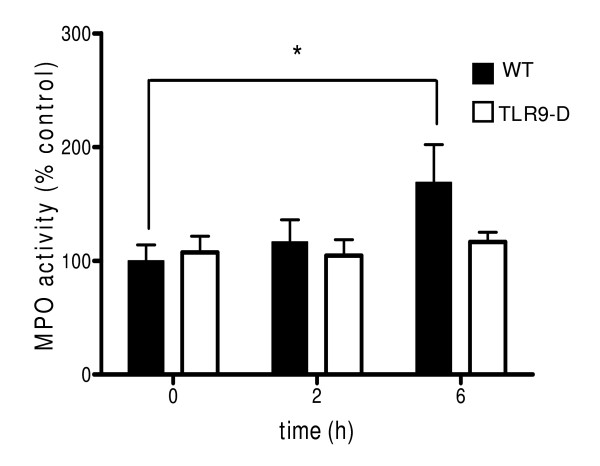
**Lung MPO content**. Content of lung MPO was documented in WT mice following application of CpG-ODN. In WT mice, MPO increased significantly 6 hrs after CpG-ODN stimulation, whereas TLR9-D mice exhibited no increase in MPO activity. Data are expressed as a % of controls (mean ± SEM; * *p *< 0.05).

In WT mice, MPO increased significantly 6 hrs after i.p. CpG-ODN stimulation. This effect was not detectable in TLR9-D mice (Figure 6).

### Bronchoalveolar lavage (BAL) after CpG-ODN stimulation

**Figure 7:**
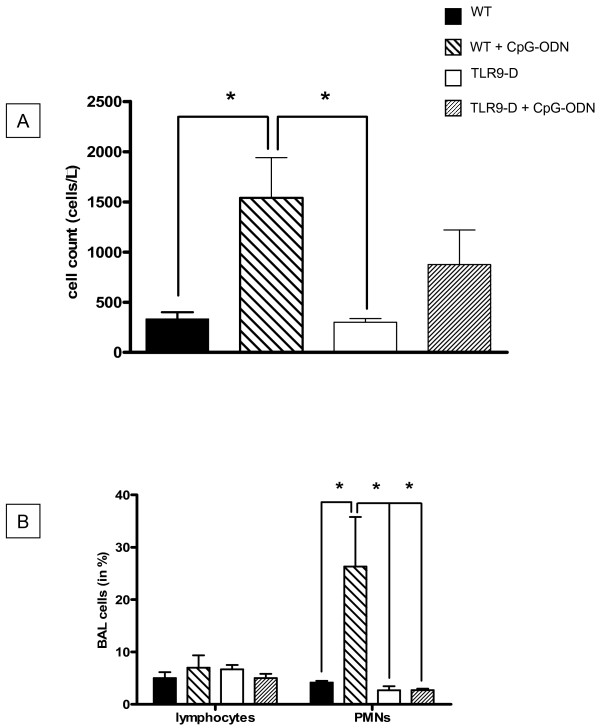
**Total and differential cell counts in BAL fluids**. WT and TLR9-D mice were challenged i.p with. CpG-ODN or saline control for 4 hrs. After CpG-ODN challenge total cell counts were significantly higher in WT mice compared to TLR9-D animals (A). In WT animals a significant increase of neutrophils (PMNs) after CpG-ODN stimulation was observed (B), which was absent in TLR9-D mice (mean ± SEM; * *p *< 0.05).

BALs demonstrated a significant increase in total cell number as well as the number of recruited neutrophils after CpG-stimulation in WT animals (Figure 7), which was diminished in TLR9-D mice. BALs obtained from all animal groups were not contaminated by peripheral blood cells indicating cell migration into the lungs.

### Leukocyte count

Under base line conditions, only a few leukocytes were detectable in both genotypes (WT: 212 ± 25 leukocytes/mm^2^; TLR9-D: 218 ± 34 leukocytes/mm^2^). 6 hrs after intratracheal stimulation, leukocyte accumulation was induced in both mouse strains. However, the detectable levels in the lungs of WT mice were significantly higher than those of TLR9-D animals (n = 5/group; 9465 ± 689 vs. 3509 ± 55 leukocytes/mm^2^, p < 0,05).

## Discussion

Acute lung injury represents acute hypoxemic respiratory failure and is associated with pulmonary and non-pulmonary risk factors. Interestingly, direct lung injury caused by bacteria and indirect lung injury associated with sepsis share similar pathophysiological pathways.

The initial host's defense against bacterial infections is essentially executed by pattern-recognition receptors. TLR 2, 4 and 5 have been implicated in bacterial signaling, innate immunity and lung inflammation [40-44]. Little is known about the role of TLR9 in the lung, but constitutive expression levels have been detected in mouse lung endothelial cells, mouse RAW264.7 cells, rat pulmonary microvascular endothelial cells and rat pulmonary artery endothelial cells [15]. High TLR9 expression levels have been found in lung tumors [15,32,33,45]. Interestingly, TLR9 is not expressed in all cells present in the lung. For instance, TLR9 is absent in rat pulmonary arterial smooth muscle cells [15], mouse pulmonary macrophages [46] and in lung dendritic cells [47]. This is in conflict with other reports demonstrating the existence of TLR9 in lung dendritic cells [46-48].

It is thought that TLR9 is able to enhance the uptake of long-chain double-stranded (ds) DNA, although single-stranded (ss) CpG-ODNs appear to be sequence-independently endocytosed. TLR9 is localized in the endoplasmatic reticulum and following CpG stimulation recruited to endosomal vesicles. Then, TLR9 and CpG-ODN co-localize resulting in cell activation [49,50]. The exact molecular structure of TLR9 is unknown, although some evidence exists that leucine-rich repeats are responsible for the recognition of distinct pathogen structures by TLRs. Following CpG-ODN binding, TLR9 associates with the adaptor molecule MyD88 resulting in activation of the IL-1 receptor-associated kinase (IRAK) family, mitogen activated kinases (MAPK), or IFN regulatory factors. The latter events activate NFκB among other transcription factors (for detailed review please refer to [51]).

Our study demonstrates a TLR9-dependent mechanism of lung inflammation. This is supported by the finding that an intraperitoneal application of CpG-ODN (extrapulmonary stimulus) leads to a systemic and local inflammatory response in WT mice, which was abolished in TLR9-D animals. Our data are in accordance with others that TLR9 is expressed in homogenisates of pulmonary tissue [15,32,33]. In addition, we observed that CpG-ODN challenge did not significantly change TLR9 expression over time. In gram-negative sepsis TLR4 expression in murine lungs did also not change; however, the expression of CD14, a co-receptor of TLR4, was up-regulated [44]. This may indicate that TLRs are differentially regulated. It is known that TLR9 stimulation leads to the activation of NFκB in various tissues [51]. To our knowledge, our study shows for the first time that pulmonary NFκB activity is up-regulated following CpG-ODN application. This is further supported by the observation that NFκB is not activated in TLR9-D animals upon CpG-ODN stimulation. In addition, CpG-ODN led to a significant increase of NFκB-dependent, proinflammatory cytokine expression (TNF-α, IL-1β) in pulmonary tissue. However, CpG-ODN did not induce an inflammatory response in TLR9-D mice indicating a TLR9-dependency. In correspondence with the presented gene expression of proinflammatory cytokines, the protein expression of TNF-α and IL-1β was significantly higher in WT animals when compared to TLR9-D mice. Furthermore, plasma levels of TNF-α and IL-6 indicate systemic inflammation in WT animals. In contrast, levels of these cytokines did not change in TLR9-D mice after CpG-challenge. This further supports our concept that CpG-ODN mediates its proinflammatory effects via TLR9. In a small pilot study we could confirm findings from others [34,52] that local (intratracheally) CpG-ODN administration also caused an inflammatory response in the lung (pulmonary stimulus), which was absent in TLR9-D mice. These findings suggest that CpG-ODN-induced lung inflammation can be initiated by both, local and systemic TLR9 activation.

Increased content of lung myeloperoxidase activity, an indicator of polymorphonuclear cells (PMNs) accumulation, was documented in WT mice following application of CpG-ODN. In WT mice, MPO increased significantly 6 hrs after CpG-ODN stimulation, whereas TLR9-D mice exhibited no increase in MPO activity. To further characterize the cellular recruitment in the pulmonary system after CpG-ODN induced inflammation a series of BALs were carried out. Since PMNs are rarely found in BAL from normal pathogen-free mice, we used this cell type as an inflammatory marker. We found a significant induction of total cell count in WT mice after CpG-ODN challenge. In particular, neutrophil counts were induced in the BAL of WT mice compared to TLR9-D animals. BALs obtained from all animal groups were not contaminated by peripheral blood indicating migration as the underlying factor. These data suggest that a significant recruitment of inflammatory cells into the alveolar space occurs after CpG-ODN stimulation.

Our findings suggest that CpG-ODN induces an inflammatory response via TLR9. In an in vivo setting of inflammation it is unlikely that bacterial DNA acts as the sole virulence factor. Other pathogenic ligands such as lipopolysaccharide and flagellin will contribute to the induction of inflammation. Recent studies have demonstrated that other TLRs and their respective ligands are also responsible for pulmonary cytokine production and pulmonary injury [42,43,53]. However, it remains unclear to what extent single virulence factors contribute to an inflammatory response. Further studies will be necessary to solve this issue.

## Conclusion

In summary, we demonstrate that CpG-ODN causes NFκB activation, leading to the induction of various cytokines in the lung and plasma and finally lung inflammation. These effects were absent in TLR9-D mice. We propose the TLR9 signalling cascade as an additional pathway to induce pulmonary inflammation.

## Competing interests

The author(s) declare that they have no competing interests.

## Authors' contributions

PK and GB conceived the study and participated in its design and coordination, both performed RNAse protection assay as well as ELISA. AK measured the MPO activitiy. MS carried out the molecular genetic studies, the i.p. injections, the sampling of the organs, Western blotting as well as RT-PCR. MV was responsible for performing the electromobility shift assay. HE performed RNAse protection assay and in particular the densitometric analysis. JM performed the leukocyte count after intratracheal installation. RM participated in the design of the study and contributed to the generation of the manuscript including the statistical analysis. AH participated in its design and coordination and helped to draft the manuscript. KZ carried out the measurement of serum cytokine levels; CG was in charge of the bronchoalveolar lavage (BAL) and cell counts. All authors read and approved the final manuscript.
